# Draft genome sequence of *Agarivorans aestuarii* strain ZMCS4, a putative CAZyme-producing bacteria isolated from the marine brown algae *Cladostephus spongiosus*

**DOI:** 10.1128/mra.01178-23

**Published:** 2024-03-27

**Authors:** Beatriz Lorente, Carla Cabral, Jorge Frias, João Faria, Duarte Toubarro

**Affiliations:** ^1^CIBIO – Research Centre in Biodiversity and Genetic Resources, InBio Associate Laboratory, University of the Azores, Ponta Delgada, Portugal; 2 Biotechnology Centre of Azores (CBA), University of the Azores, Ponta Delgada, Portugal; DOE Joint Genome Institute, Berkeley, California, USA

**Keywords:** *Agarivorans aestuarii*, CAZymes (carbohydrate-active enzymes), genome sequencing, marine microbiology

## Abstract

We report the draft genome sequence of *Agarivorans aestuarii* strain ZMCS4, isolated from *Cladostephus spongiosus*. The assembled genome consists of 4.5 Mbp, comprising 25 contigs and 4,128 coding sequences. This genome will provide insights into further studies on relevant CAZymes involved in the hydrolysis of algal cell walls.

## ANNOUNCEMENT

The genus *Agarivorans* was initially proposed by Kurahashi and Yokota in 2004 (1).Currently, this genus comprises four species, all of which have been isolated from various marine environments, including marine animals, seawater, marine sediments, and seaweeds (1–4). The major characteristics among *Agarivorans* spp. are the agarolytic activity and seaweed polysaccharides degradation activity; this likely plays a role for alga-feeding marine creatures and within the carbon cycle of the marine ecosystem (1,5, 6).

During the summer of 2019, a collection of macroalgae samples was conducted in a marine shallow water CO_2_ seep site (~5 m depth) on São Miguel Island in the Azores, Portugal (coordinates 37.727113,–25.316208) (7, 8). The seaweeds were rinsed three times with sterilized filtered sea water. Samples were homogenized, serially diluted, and cultivated on Marine Broth 2216 agar plates at 20°C ± 2°C for 72 h exposed to natural light-dark cycle. A white colony with high agar-degrading activity, associated to the brown algae *Cladostephus spongiosus* (Hudson) C.Agardh, 1817, was cultured. Single colonies were picked and restreaked in order to obtain a pure culture. The strain was cryopreserved and given the designation of ZMCS4. To extract the genomic DNA, strain ZMCS4 was cultured for 48 h at 25°C at 180 rpm in Marine Broth 2216, and the cells were harvested by centrifugation. DNeasy PowerSoil Kit (Qiagen) was used, according to the manufacturer’s instructions. The 16S rRNA gene was amplified using the universal primers 8F and 1492R (9) to analyze the taxonomic position. The PCR product was purified and sent for sequencing to StabVida (Lisbon, Portugal). A partial sequence of 16S rRNA gene was obtained (1,358 bp), and the comparison via NCBI-BLAST indicates that the strain ZMCS4 belongs to the genus *Agarivorans*, and it exhibited the highest similarity (98.97%) to that of *Agarivorans aestuarii* hydD622 (GenBank: NR_151931.1) and to *Agarivorans aestuarii* KCTC 32543 (GenBank: AP023033.1) (98.90%).

DNA libraries were prepared from the same genomic DNA extract, with a TruSeq SBS Kit v5 (Illumina) protocol, and the genome was sequenced using NovaSeq 6000 system, with paired-end read sizes of 150 bp. A total of 7,648,242 paired-end reads, resulting in an average depth coverage of 245×, were obtained. Raw sequences were trimmed (ambiguous limit: 2nucleotides; quality limit: 0.01 error; minimum number of nucleotides in reads: 30 nucleotides) and used for the *de novo* assembly using CLC Genomics Workbench 12.0.3 (10). QUAST v.5.0.2 (11) was used to perform a quality assessment and evaluation of the genome assembly. The assembled draft genome was annotated using the PGAP pipeline from NCBI (12), additionally the genes encoding for CAZymes were detected using HMMER v3.3.2 package (http://hmmer.org/) with the dbCAN CAZyme database (13).

The assembled draft genome of *Agarivorans aestuarii* strain ZMCS4 consists of 25 contigs with a total length of 4,570,580 bp (44.67% C + G contents), the N50 contig size is 308,937 bp, and the longest contig is 869,673 bp. Genome annotation resulted in 4,128 coding sequences. The genome includes 363 sequences encoding for 194 CAZyme families, mainly belonging to glycoside hydrolases (mostly GH5, GH13, and GH16) and polysaccharide lyases (mostly PL7 and PL12), 64% and 7%, respectively (Additional file 1). These CAZYme families are involved in the hydrolysis of agar, alginate, carrageenan, and other polysaccharides that are abundant in seaweeds.

## Data Availability

The raw sequencing data (SRR28237126) and the draft genome (accession number JAYDYW000000000) have been deposited in NCBI portal, under de BioProject PRJNA1045876. The partial sequence of the 16S rRNA gene was deposited in Genbank under accession number MZ555776. The strain ZMCS4 was deposited at the DSMZ (German Collection of Microorganisms and Cell Cultures) under the reference number DSM 113257. The Additional file 1, with the predicted CAZyme genes of Agarivorans aestuarii strain ZMCS4, is available at Figshare https://doi.org/10.6084/m9.figshare.25342222.v1.
